# Interconnected Hierarchically Porous Graphene‐Based Membrane Electrode for High‐Power and Long‐Cycle Lithium–Oxygen Battery

**DOI:** 10.1002/advs.202519091

**Published:** 2025-12-01

**Authors:** Arghya Dutta, Takashi Kameda, Taiga Ozawa, Anna Myojin, Minako Nishioka, Wei Yu, Hirotomo Nishihara, Shoichi Matsuda

**Affiliations:** ^1^ Center for Green Research on Energy and Environmental Materials National Institute for Materials Science 1‐1 Namiki, Tsukuba Ibaraki 305‐0044 Japan; ^2^ Advanced Institute for Materials Research (WPI‐AIMR) Tohoku University Sendai Miyagi 980‐8577 Japan; ^3^ Center for Advanced Battery Collaboration National Institute for Materials Science 1‐1 Namiki Tsukuba Ibaraki 305‐0044 Japan

**Keywords:** carbon electrode, high power battery, lean electrolyte, lithium–oxygen battery, porosity optimization, rechargeable battery

## Abstract

The energy–power trade‐off in lithium–oxygen batteries (LOBs) arises from sluggish oxygen (O_2_) transport in the porous positive electrode and pore clogging by lithium peroxide (Li_2_O_2_). While increasing porosity enhances electrolyte accessibility and Li_2_O_2_ storage, it also increases electrolyte demand, compromising the overall energy density of the cell and necessitating alternative strategies to boost power capabilities without sacrificing energy density. In this study, theoretical simulations of O_2_ transport reveal that reducing tortuosity by improving pore interconnectivity has a more significant impact on O_2_ transport than porosity itself. Based on this insight, a freestanding graphene‐based electrode with a highly interconnected macroporous network is fabricated via a non‐solvent‐induced phase separation approach using polyacrylonitrile (PAN) as a carbon scaffold and polyethylene oxide (PEO) as a sacrificial porogen. The selective decomposition of PEO creates spatially interconnected macropores, effectively reducing tortuosity. The resulting electrode enables LOB cells to achieve >2500 mAh g^−1^ at 1.0 mA cm^−2^ under lean‐electrolyte conditions. Stable cycling at 4 mAh cm^−2^ is maintained with only 3.25 g Ah^−1^ electrolyte, and high‐rate performance persists over 90 cycles at 1.5 mA cm^−2^. This work demonstrates a robust strategy to simultaneously improve energy and power performance in practical LOBs through rational electrode architecture.

## Graphical abstract

Lithium–oxygen batteries face an energy‐power trade‐off due to sluggish O_2_ transport and pore clogging by Li_2_O_2_. Simulations show that reducing tortuosity via interconnected macropores enhances oxygen mobility. A freestanding graphene electrode having interconnected macropores with low tortuosity delivers >2500 mAh g^−1^, stable lean‐electrolyte cycling, and high‐rate performance, providing a practical strategy to improve both energy and power in LOBs.

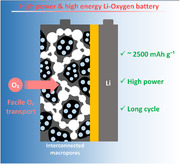

## 1 Introduction

Lithium–oxygen batteries (LOBs) have garnered significant interest as a next‐generation energy storage system due to their exceptionally high theoretical gravimetric energy density (≈3500 Wh kg^−1^), far surpassing conventional lithium‐ion batteries (LIBs).^[^ ^1^, ^2^ ^]^ However, despite their promise, the practical realization of LOBs is hindered by several critical challenges, including lower‐than‐expected specific capacity, poor power performance, and limited cycle life.^[^ ^3^, ^4^, ^5^, ^6^, ^7^ ^]^ These challenges primarily stem from the fundamental electrochemical processes and the structural limitations of LOB components. A typical LOB consists of a lithium (Li) metal negative electrode, a non‐aqueous electrolyte, and a porous carbon‐based positive electrode. During discharge, lithium ions (Li⁺) react with oxygen (O_2_) to form solid lithium peroxide (Li_2_O_2_), which is subsequently decomposed during the charge cycle (2Li⁺ + O_2_ + 2e^−^ ↔ Li_2_O_2_). However, the accumulation of insoluble Li_2_O_2_ within the porous carbon obstructs ion and O_2_ transport, leading to electrode passivation, lower‐than‐expected capacity, large overpotential, poor rate capability, and short cycle life.^[^ ^8^, ^9^, ^10^ ^]^ Therefore, designing an optimized carbon structure that facilitates effective Li_2_O_2_ deposition and decomposition while maintaining open pathways for ion and gas diffusion is crucial for enhancing LOB performance. Extensive investigations have highlighted the critical role of carbon electrode architecture in governing the discharge behavior and overall performance of LOBs.^[^ ^11^, ^12^, ^13^, ^14^, ^15^, ^16^, ^17^, ^18^, ^19^ ^]^ A consensus has emerged that an ideal air electrode must consist of three structural features: i) a large surface area to maximize electrochemical reaction sites, ii) sufficiently wide pores to facilitate efficient O_2_ and Li⁺‐ion transport while preventing pore clogging, and iii) a high pore volume to accommodate the substantial formation of solid Li_2_O_2_ discharge products without severely compromising performance.

While considerable progress has been made in tuning the porosity of carbon electrodes for LOBs, several critical aspects, such as the spatial interconnectivity of pores, the packing of carbon particles, and their implications for energy and power densities, have remained largely underexplored. Traditional design strategies focus primarily on increasing electrode porosity to enhance O_2_ transport and boost discharge capacity.^[^ ^14^, ^15^, ^16^ ^]^ However, highly porous electrodes require substantial volumes of electrolyte to ensure adequate wetting.^[^ ^20^, ^21^ ^]^ This increased electrolyte demand significantly compromises the cell‐level energy density, posing a major challenge to the practical deployment of LOBs. Despite its importance, the role of electrolyte volume has received limited attention in the field. Most reported systems still operate with excess electrolyte (>50 µL cm^−2^) and low areal capacities (<1 mAh cm^−2^), resulting in cell‐level energy densities that fall short of those achieved in commercial LIBs.^[^ ^22^, ^23^ ^]^ Furthermore, conventional porous electrodes are typically fabricated using polymeric binders, which promote dense particle aggregation. This compact microstructure limits O_2_ diffusivity and reduces the accessible pore volume for Li_2_O_2_ growth, leading to low active material utilization, limited capacity, and poor rate performance. In addition, the presence of binders can introduce parasitic reactions that further deteriorate battery stability.^[^ ^24^, ^25^ ^]^ To overcome these limitations, the development of binder‐free, self‐supporting carbon architectures with engineered porosity and improved pore interconnectivity (low tortuosity) is highly desirable. Such designs aim to simultaneously enhance O_2_ transport pathways and facilitate more efficient Li_2_O_2_ formation, thereby improving both energy and power performance in practical LOB systems.

Beyond structural optimization, the surface chemistry of carbon electrodes plays a crucial role in determining LOB stability. Highly graphitized carbons with minimal surface functional groups exhibit superior long‐term cycling performance by reducing side reactions with reactive oxygen species.^[^ ^23^, ^26^ ^]^ Consequently, significant research efforts have been directed toward enhancing the degree of graphitization and crystallinity of carbon materials. Our recent works introduced edge‐site‐free graphene electrodes with hierarchical porosity, achieving high capacity and extended cycle life.^[^ ^27^, ^28^ ^]^ The mesoporous framework was created using aluminum oxide (Al_2_O_3_) nanoparticles as hard templates, while macropores formed between spherical carbon aggregates. However, the absence of a well‐interconnected long‐range macroporous network and unoptimized porosity resulted in limited rate capability and excessive electrolyte dependence to maintain long cycling stability.

Based on these considerations, at first, we theoretically simulate the effects of porosity and the pore‐connectivity (tortuosity) on O_2_ diffusion through the porous positive electrode. Then, we focus on developing spatially interconnected hierarchically porous self‐standing graphene mesosponge (GMS) membranes optimized for high capacity, superior rate performance, and stable cycling under lean electrolyte conditions. The highly interconnected meso‐macroporous network provides efficient O_2_ and ion diffusion pathways while offering sufficient space for Li_2_O_2_ deposition. The electrode fabrication process involved three steps: First, a porous GMS carbon was synthesized, enabling modulation of structural properties for improved electrochemical stability and porosity in different dimensions, from micropore to mesopore. Second, self‐standing membranes were fabricated using the doctor blade technique with a carbon‐based slurry containing polyacrylonitrile (PAN) and polyethylene oxide (PEO). The non‐solvent‐induced phase separation (NIPS) method introduced a macroporous network into the polymer matrix. The third step employed the carbonization of the hierarchically porous membrane to convert the polymer matrix into macroporous carbon. The differences in the thermal stabilities of PAN and PEO were leveraged to regulate the macroporous network. During carbonization, the PAN matrix transformed into an isolated macroporous carbon scaffold, whereas PEO decomposed into low‐molecular‐weight non‐solid products, opening up the porous network and producing an interconnected macropore structure. The formation of this interconnected macroporous network is crucial to achieving high‐power operation of an LOB. This hierarchical design balances meso‐ and macroporosity, optimizing electrode capacity and electrolyte requirements for sufficient wetting under lean electrolyte conditions.

## 2 Results and Discussions

### 2.1 Simulation of Quantitative Impacts of Porosity and Pore Connectivity (Tortuosity) on the Oxygen Diffusion

**Figure 1:**
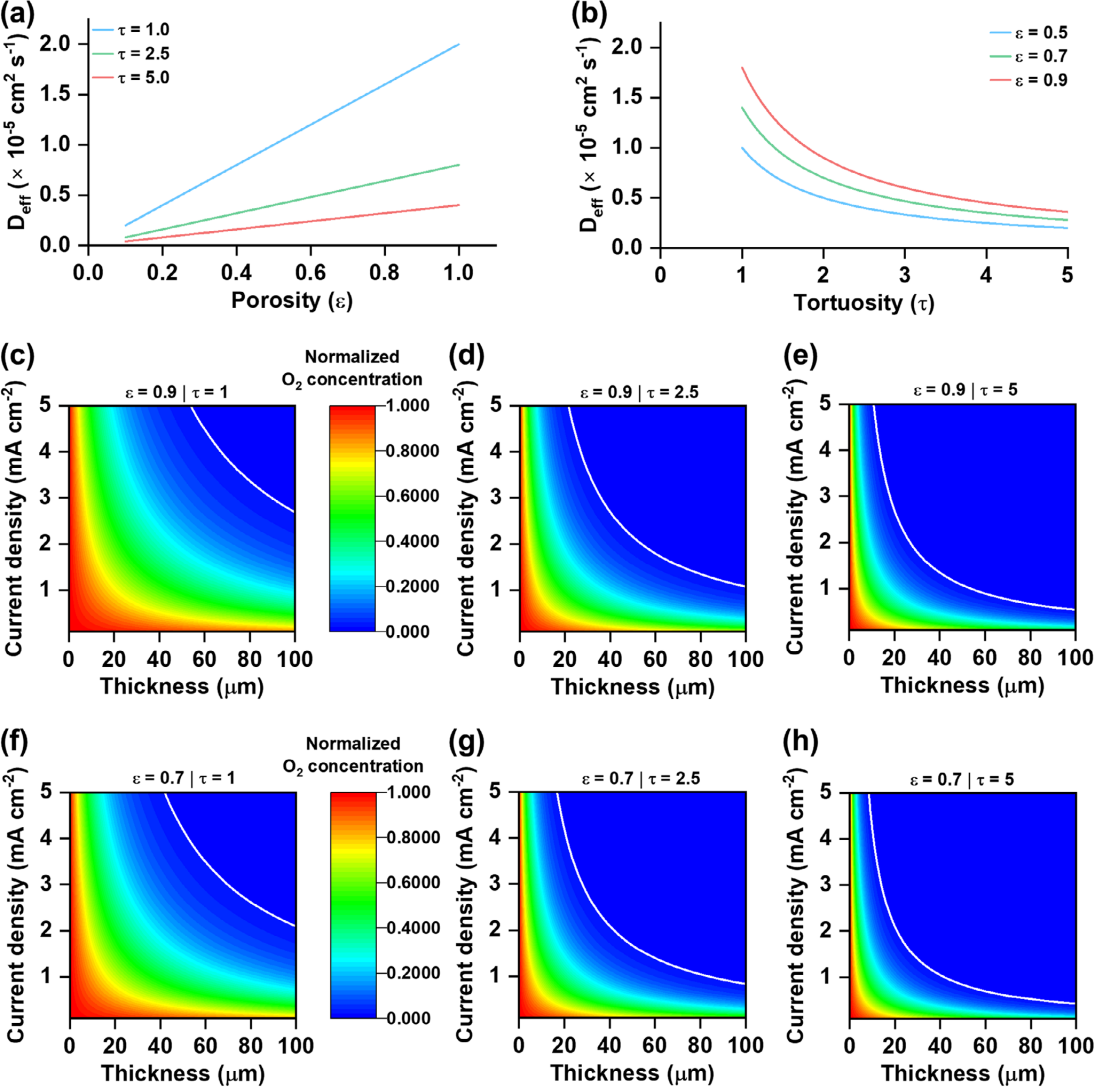
Variation of the effective diffusion coefficient (*D*_eff_) of oxygen (O_2_) with a) porosity (*ε*) and b) tortuosity (*τ*). c–h) Contour plots of normalized O_2_ concentration across the electrode thickness under different current densities.

During the discharge process of LOBs, molecular O_2_ dissolved in the liquid electrolyte diffuses into the porous positive electrode, where it undergoes electrochemical reduction. This reduction process is often modeled as a first‐order reaction with respect to the local O_2_ concentration, and it is strongly influenced by the balance between O_2_ supply via diffusion and its electrochemical consumption.^[^ ^29^ ^]^ Under constant current (galvanostatic) operation, the applied current density dictates the rate at which O_2_ must be reduced, which in turn requires a steady diffusive flux of O_2_ across the porous structure of the electrode. If this flux is insufficient to meet the electrochemical demand, O_2_ concentration gradients develop, ultimately leading to mass transport limitations. Therefore, the rate capability or the power density of the LOB cell directly depends on the efficiency of O_2_ diffusion in the porous positive electrode. In this regard, the architecture of the porous positive electrode plays a crucial role in determining the efficiency of O_2_ transport to reaction sites. Two microstructural parameters, porosity (ε) and tortuosity (τ), are especially important. Porosity is defined as the fraction of void volume within the total electrode volume and relates to the space available for electrolyte infiltration and gas transport. A higher porosity generally provides more open channels for O_2_ diffusion. Tortuosity, on the other hand, characterizes the geometric complexity and connectivity of the pore network. It quantifies how much longer or more convoluted the actual diffusion path is compared to a straight‐line path across the electrode. Increased tortuosity results in greater resistance to mass transport due to effects such as dead‐end pores, narrow necks, and poorly connected pathways. The effective diffusion coefficient (*D*_eff_) captures how these two parameters modulate O_2_ transport in the electrode and is typically expressed as:^[^ ^30^ ^]^

(1) $$D_{\mathrm{eff}}=D_{0}\frac{\varepsilon }{\tau }$$

where *D*_0_ is the bulk diffusion coefficient of O_2_ in the electrolyte. This relationship makes clear that while increasing porosity facilitates diffusion, increasing tortuosity significantly impedes it. **Figure** **1**a,b illustrates the dependence of *D*_eff_ on porosity and tortuosity, respectively. As shown in Figure 1a, higher porosity leads to a linear increase in *D*_eff_, as more free volume becomes available for O_2_ molecules. In contrast, Figure 1b highlights a stronger non‐linear inverse dependence of *D*_eff_ on tortuosity, where small increases in τ can lead to a substantial reduction in effective diffusivity.

To quantitatively evaluate how the microstructure of the positive electrode, specifically ε and τ, influences O_2_ availability, we modeled steady‐state 1D diffusion across the electrode thickness, incorporating a homogeneous first‐order reaction term, by the following equation:^[^ ^21^ ^]^

(2) $$C\left(x\right)=C_{0}\exp\left(-x\frac{j}{nFC_{0}D_{\mathrm{eff}}}\right)$$

where *C*(*x*), *C*_0_, *j*, *n*, *F*, and *D*_eff_ are the concentration of O_2_ in the electrode at distance *x* from electrode‐O_2_ interface, O_2_ solubility in the bulk electrolyte, current density normalized to the electrochemically active surface area (ECSA) of the carbon, number of electrons in the rate limiting step, Faraday constant, and effective diffusion coefficient of O_2_ in the electrolyte inside the porous electrode, respectively. The value of *n* is taken to be 1, based on previous reports, considering one electron reduction step (O_2_ + e^−^ ↔ O_2−_) to be kinetically rate‐limiting during the discharge of LOBs.^[^ ^29^, ^31^, ^32^ ^]^ This framework captures the critical coupling between structural parameters and electrochemical kinetics under various discharge conditions. Figure 1c–h displays normalized O_2_ concentration (*C*(*x*)/*C*_0_) profiles as contour plots across the electrode thickness, resolved for a matrix of porosity values (*ε* = 0.9 and 0.7) and tortuosity factors (*τ* = 1, 2.5, and 5). These parameter sets represent idealized open structures to more restrictive, highly convoluted diffusion pathways, respectively. At high porosity (*ε* = 0.9), the diffusion pathways offer substantial void space for gas‐phase transport. In the ideal case with minimal tortuosity (*τ* = 1, Figure 1c), > 25% of the initially available O_2_ is efficiently delivered throughout the electrode depth of 100 µm, even at high current densities approaching 1.0 mA cm^−2^. However, as *τ* increases to 2.5 and 5 (Figure 1d,e), the effective diffusivity decreases due to more tortuous transport paths. Consequently, under τ = 5, O_2_ becomes significantly depleted before reaching the separator side, with < 2.5% of initial O_2_ concentrations observed at 1.0 mA cm^−2^, marking a clear transition to diffusion‐limited behavior. The effect of reduced porosity (*ε* = 0.7) further exacerbates the limitations on O_2_ transport. Even under favorable tortuosity (τ = 1, Figure 1f), the O_2_ profile shows markedly decreased concentration at current density 1.0 mA cm^−2^. Only 15% of the initial O_2_ concentration can be transported through the thickness of 100 µm at 1.0 mA cm^−2^. When τ is increased to 2.5 and 5 (Figure 1g,h), these O_2_ concentration values drop sharply. In particular, at ε = 0.7 and τ = 5, O_2_ is almost entirely depleted near the separator at discharge rates above 0.5 mA cm^−2^, leaving much of the electrode underutilized due to insufficient reactant supply. The solid white lines in the contour plots indicate regions where the O_2_ concentration drops to 2% of its initial value. As porosity decreases and tortuosity increases, these white lines shift toward lower electrode thicknesses and current densities, underscoring the increasing limitation on O_2_ transport under less favorable structural conditions. These simulations clearly demonstrate that achieving high porosity alone is insufficient to ensure effective O_2_ transport at practical discharge rates in LOBs. Our simulations suggest that lowering tortuosity could be a more effective strategy to improve O_2_ transport and enable higher power performance in LOBs, provided that such control can be realized experimentally.

### 2.2 Design Strategy of Spatially Interconnected Graphene‐Based Porous Electrode

**Figure 2:**
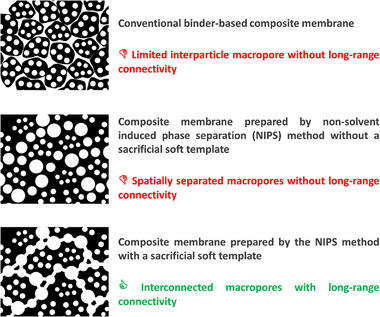
Schematic illustration of the structural differences among various carbon electrodes depending on the fabrication approach. The top panel depicts a particle‐based porous carbon electrode fabricated using a binder, where pores are primarily confined within individual particles. The middle panel shows a nonideal meso‐macroporous carbon membrane in which macropores exist but remain spatially isolated, limiting mass transport. The bottom panel illustrates the ideal architecture, a freestanding carbon membrane featuring a continuous network of interconnected macropores that facilitate efficient electrolyte infiltration and gas diffusion. Small white circles denote mesopores, while large white circles indicate macropore. The micropores and interconnectivity through microporous channels are not shown.

Despite significant efforts to tailor carbon porosity, the spatial organization of carbon within the electrode and the tortuosity factor remain largely overlooked. Conventional electrodes, composed of carbon particles bound by polymers, often form densely packed structures that restrict O_2_ diffusion and limit active material utilization.^[^ ^33^ ^]^ **Figure** **2** schematically illustrates, through extreme hypothetical examples, the contrast between ideal and nonideal electrode architectures. The top panel shows conventional electrodes with poor pore connectivity, absence of macropores, and blocked transport channels. The middle panel represents a nonideal structure with macropores lacking interconnectivity, thus consuming a high volume of electrolyte without improving mass transport. The bottom panel illustrates the optimally ideal case: a network of interconnected macropores that supports efficient gas diffusion and electrolyte transport.

**Figure 3:**
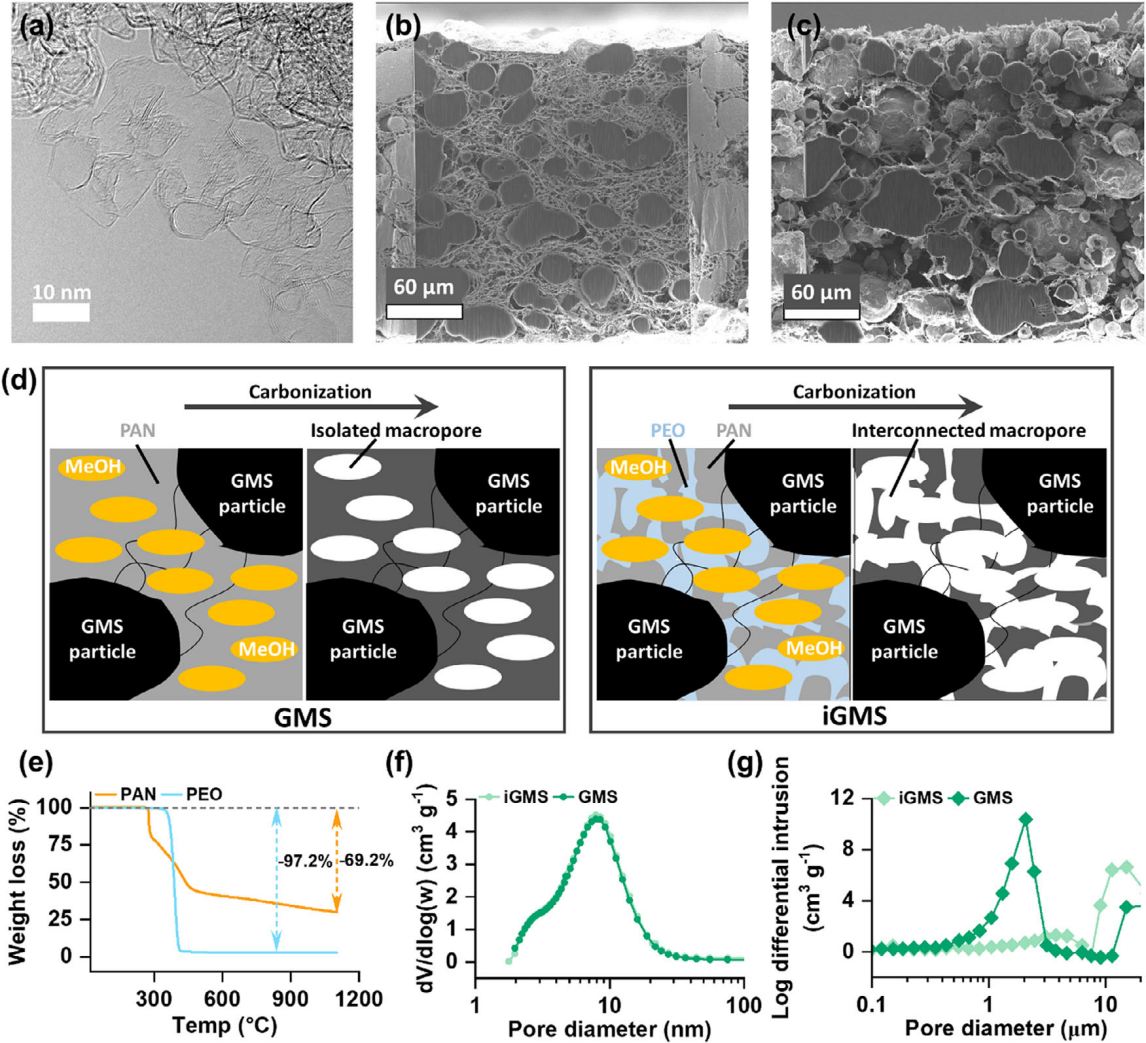
a) Transmission electron micrograph (TEM) of GMS powder sample. Cross‐section scanning electron micrograph (SEM) of b) GMS and c) iGMS membranes. d) Schematic representation of the electrode fabrication processes following NIPS method with and without using PEO. e) Thermogravimetric analysis (TGA) data of PAN and PEO under a helium atmosphere. Pore size distribution curves of GMS and iGMS membranes measured by f) N_2_ adsorption/desorption and g) Hg porosimetry.

Motivated by this understanding, we developed a freestanding graphene‐based electrode with a hierarchically interconnected porous framework. A GMS carbon was synthesized based on a previously reported method.^[^ ^34^ ^]^ The scanning electron micrograph (SEM) in Figure S1 (Supporting Information) reveals a spherical morphology of the GMS particles. The particle‐size distribution curve of the GMS, derived from SEM analysis and shown in Figure S2 (Supporting Information), indicates a uniform size distribution centered around 50 µm. The transmission electron micrograph (TEM) in **Figure** **3**a shows that the GMS particles are actually aggregates of graphene flakes. The structural and surface chemical properties of the GMS carbon were thoroughly characterized using X‐ray diffraction (XRD), Raman spectroscopy, and X‐ray photoelectron spectroscopy (XPS), and the results were compared with those of the commercial KB carbon. The results from XRD patterns for GMS and KB powder samples are shown in Figure S3 (Supporting Information). A comparison of the Raman spectra, shown in Figure S4 (Supporting Information), also reveals that GMS exhibits comparatively sharper peaks, and a lower *I*_D_/*I*_G_ ratio (1.62 vs 1.82 for KB) in Figure S5 (Supporting Information) confirms a lower structural defect in GMS. Surface chemical analysis via XPS reveals slightly higher carbon content in GMS compared to KB, as shown in Figure S6 (Supporting Information). Nevertheless, both carbons exhibit similar types of oxygen‐containing functional groups. Deconvoluted XPS spectra (Figures S7–S10, Supporting Information) identify C1s peaks at 285.2–285.6, 286.8–287.0, and 289.8–290.2 eV, which correspond to C−O, C═O, and COO^−^ functional groups, respectively, with corresponding O1s peaks at 533.2–533.6 eV (C−O), 532.0–532.5 eV (C═O), and 535.5–535.6 eV (COO^−^).^[^ ^23^ ^]^ These results indicate that the GMS electrode possesses enhanced graphitization and a higher carbon content, which are expected to enhance the electrochemical stability of GMS‐based electrodes relative to KB electrodes.

As mentioned previously, the porous carbon positive electrode in LOBs should have high O_2_ diffusion ability and efficient electrochemical properties. From a practical applicability and scalability point of view, a self‐standing hierarchically porous electrode is a desirable choice. Therefore, self‐standing carbon membranes were fabricated using a slurry casting technique combined with NIPS process, developed within our research group.^[^ ^35^ ^]^ The slurry was prepared by mixing carbon powders, carbon nanotubes (CNTs), PAN, and/or PEO in N‐methyl‐2‐pyrrolidone (NMP) solvent. CNTs were incorporated to enhance the mechanical integrity of the membranes. A uniform carbon film was prepared using a doctor blade technique and subsequently immersed in methanol, a poor solvent, to initiate the NIPS process, leading to the development of macroporous voids within the film. The membranes were dried and then underwent heat treatment followed by carbonization at 1050 °C under an inert atmosphere. During the carbonization process, PAN transformed into a robust carbon scaffold with isolated macropores, while PEO decomposed into volatile compounds, opening up the macroporous channels and forming an interconnected hierarchical macro‐mesoporous network.^[^ ^36^, ^37^ ^]^ The sacrificial decomposition of PEO played a pivotal role in connecting the macropores, enabling efficient transport of O_2_ dissolved in the electrolyte. The cross‐sectional focused ion beam (FIB) SEM image in Figure 3b reveals that the GMS/PAN electrode without PEO (denoted as GMS) displays the formation of robust macropores, which appear to be isolated without extended connection via macropores. These macropores can be linked through microporous walls that permit electrolyte infiltration, but such connections via micropores provide limited pathways for efficient mass transport during electrochemical reactions. However, in contrast, when PEO was incorporated, as shown in the FIB‐SEM image in Figure 3c, the non‐oxidative thermal decomposition of PEO created open channels through the pore walls and established an extended network of interconnected macropores in the electrode (denoted as iGMS). A proposed mechanism of these two types of electrode preparation is shown in Figure 3d. The thermogravimetric analysis (TGA) data for PAN and PEO in Figure 3e under an inert atmosphere show that upon heating to 1100 °C, PAN exhibited a 69.2% mass loss, while PEO lost 97.2% of its mass. These results confirm the carbonization of PAN and the non‐oxidative decomposition of PEO.

We performed a series of structural characterizations for the three kinds of carbon electrodes: KB, GMS, and iGMS membranes. Figures S11 and S12 (Supporting Information) illustrate the N_2_ adsorption/desorption isotherms for the GMS and iGMS membranes, while a reference KB membrane is included for comparison in Figure S13 (Supporting Information). Brunauer−Emmett−Teller (BET) surface area analysis reveals that the addition of PEO has a negligible impact on the BET surface area of the GMS electrodes, which measured 1711 m^2^ g^−1^ with PEO and 1667 m^2^ g^−1^ without PEO. Similarly, the Barrett‐Joyner‐Halenda (BJH) pore size distribution curves (Figure 3f) indicate no difference in mesopore diameters between GMS electrodes fabricated with and without PEO. Detailed analysis (**Table** **1**) shows that the iGMS electrode also exhibits nearly similar mesopore volumes (2.8 cm^3^ g^−1^) compared to the GMS electrode (2.73 cm^3^ g^−1^). Macropore volume analysis using mercury (Hg) porosimetry, as shown in Figure 3g, highlights significant differences in the macroporous structures of the electrodes. The macropores with diameters of approximately 2 µm in the GMS membrane were collapsed due to the sacrificial decomposition of PEO during carbonization, resulting in a reduction in macropore volume from 3.28 cm^3^ g^−1^ in the GMS membrane to 1.18 cm^3^ g^−1^ in the iGMS membrane. Interestingly, the decomposition of PEO facilitated the opening of larger macropores with diameters exceeding 10 µm. These large macroporous channels are critical for enabling efficient electrolyte diffusion within the electrode, particularly for practical applications requiring thicker electrodes to achieve high specific energy. Overall, these results underscore the advantages of the NIPS process combined with the strategic selection of polymer materials, allowing precise control over the porosity of the carbon membrane electrode.

**Table 1:** Surface area and pore volume of different membrane electrodes measured by N_2_ adsorption/desorption and Hg porosimetry.

Membrane electrode	BET surface area [m^2^ g^−1^]	Pore volume [cm^3^ g^−1^]			
		<2 nm	2–20 nm	>20 nm	0.2–10 µm
GMS	1667	0.59	2.56	0.17	3.28
iGMS	1711	0.60	2.45	0.35	1.18
KB	802	0.35	2.15	2.45	2.33

### 2.3 Application of GMS Electrodes in LOBs

**Figure 4:**
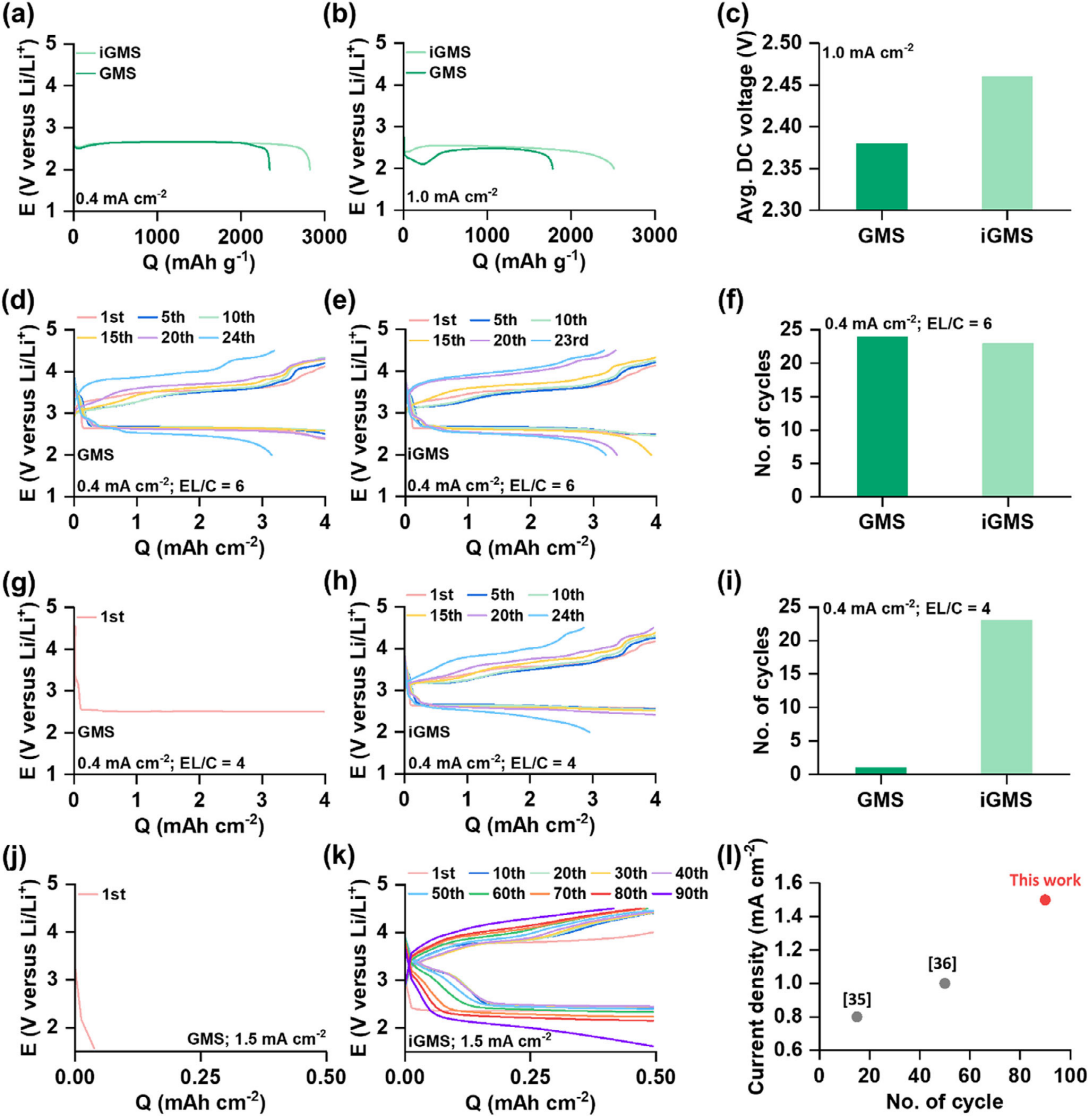
Galvanostatic discharge profiles of LOB cells with GMS and iGMS electrodes at a) 0.4 mA cm^−2^ and b) 1.0 mA cm^−2^ up to 2.0 V versus Li/Li⁺. c) Average discharge voltages at 1.0 mA cm^−2^. Cycling profiles of d) GMS and e) iGMS at 0.4 mA cm^−2^ with 4 mAh cm^−2^ capacity under EL/C = 6, and f) corresponding cycle lives. Cycling profiles of g) GMS and h) iGMS under EL/C = 4, and i) corresponding cycle lives. High‐rate cycling of j) GMS and k) iGMS electrodes. l) Comparison of high‐rate cycling stabilities of iGMS electrode with previously reported cells cycled at current densities ≥ 0.8 mA cm^−2^ and limited capacities.

To evaluate the impact of pore optimization on electrode performance, the discharge capacities of the electrodes were assessed in a stack‐type cell (Figure S14, Supporting Information) using a controlled amount of 1 m lithium bis(trifluoromethanesulfonyl)imide (LiTFSI) in tetraethylene glycol dimethyl ether (TEGDME or G4) as the electrolyte. The electrolyte loading amount was set to the electrolyte to carbon mass ratio (EL/C) of 5. Discharge tests were performed at current densities of 0.4 and 1.0 mA cm^−2^ to investigate the effect of pore optimization on rate‐dependent capacities. The actual membrane electrode masses, along with the mass‐normalized current density and specific capacity values, are listed in Table S1 of the Supporting Information. At a low discharge rate of 0.4 mA cm^−2^, the GMS and iGMS electrodes exhibited higher capacities of 2350 and 2850 mAh g^−1^, respectively (**Figure** **4**a), whereas the KB electrode achieved a slightly lower capacity of approximately 2100 mAh g^−1^ (Figure S15, Supporting Information). In contrast, at a higher discharge rate of 1.0 mA cm^−2^, the advantages of GMS electrodes and pore optimization become strikingly evident. As shown in Figure 4b and Figure S16 (Supporting Information), the GMS electrode achieved a significantly high capacity of 1800 mAh g^−1^, and the iGMS electrode further improved to an impressive 2510 mAh g^−1^. The concave feature observed in the initial stage of the discharge curve of GMS in Figure 4b arises from transient limitations in electrolyte transport within the porous GMS electrode under lean‐electrolyte and high‐rate conditions. As discharge proceeds, Li_2_O_2_ gradually deposits within the pores, partially blocking the pores and mitigating electrolyte scarcity, which allows the voltage to recover. The KB electrode, in comparison, exhibited a negligible capacity of just 12 mAh g^−1^ (Figure S17, Supporting Information). Moreover, Figure 4c shows that the average discharge potential of iGMS electrode (2.46 V vs Li/Li⁺) is higher than that of GMS electrode (2.38 V vs Li/Li⁺) at a high rate of 1 mA cm^−2^. It is important to mention that the capacity trends observed here inversely correlate with the total pore volume (combining meso‐ and macropores) of the electrodes: KB (6.93 cm^3^ g^−1^) > GMS (6.01 cm^3^ g^−1^) > iGMS (3.98 cm^3^ g^−1^). These results emphasize that, under lean electrolyte and high current density conditions, discharge capacity is not directly correlated with the total pore volume of the electrode. Instead, an optimized pore structure concerning efficient electrolyte diffusion and proper electrode filling is critical for achieving high capacity, particularly under fast discharge conditions. A highly interconnected porous network that promotes efficient diffusion, together with a balanced pore volume that minimizes excessive electrolyte demand while maintaining sufficient capacity, provides a viable approach to address the energy–power trade‐off in LOBs. While large pore volumes in carbon electrodes intuitively provide sufficient space for Li_2_O_2_ deposition and facilitate O_2_ and Li‐ion transport, they also require substantial electrolyte volumes for effective pore‐filling. Under lean electrolyte conditions (EL/C = 5), the large pore volume of the KB‐based electrode resulted in incomplete pore filling, leading to significant voltage polarization and low capacity at high rates. Conversely, the reduced pore volumes of GMS‐based electrodes minimized electrolyte requirements, enabling higher capacities under the same conditions. Between the GMS and iGMS electrodes, the connection of macropores and reduction of macropore volume in the iGMS electrode further improved capacity and rate capability. The enhanced electrolyte diffusion facilitated by interconnected pores (lower tortuosity) and the reduction of inactive porosity were key to this performance improvement. The impedance spectra of carbon|separator|carbon symmetric cells using GMS and iGMS electrodes with three electrolyte loadings (EL/C = 4, 5, and 6) are presented in Figures S18–S20 (Supporting Information). When the electrodes are adequately filled with electrolyte at EL/C = 5 and 6, both cells exhibit comparable impedance values. However, under lower electrolyte loading conditions (EL/C = 4), the iGMS electrode with its interconnected porous architecture displays significantly lower impedance. Because the symmetric cell configuration eliminates faradaic contributions, the measured impedance primarily reflects electrolyte transport within the porous membrane. Under lean electrolyte conditions, limitations in electrolyte diffusion and insufficient electrode filling play a more dominant role than total pore volume in determining discharge capacity, particularly during high‐power operations. The cell level energy and power density values of KB, GMS, and iGMS electrodes along with the high performing electrodes reported in the literature are compared in Figure S30 and Table S2 (Supporting Information). The masses of all the cell components including the electrolyte mass are considered for the calculation of gravimetric energy and power densities. The results reveal that for GMS‐based electrodes, particularly iGMS, increasing the power density has minimal impact on energy density. In contrast, the KB electrode exhibits a pronounced drop in energy density under high‐power operation. Furthermore, the iGMS electrode exhibits a cell level energy density of ≈990 Wh kg^−1^, which is comparable to that of several recently reported high‐energy‐density pouch‐type LOB cells at similar power densities (≈30 W kg^−1^), while maintaining excellent retention (> 800 Wh kg^−1^) under high‐power operation (≈80 W kg^−1^). These findings underscore the importance of optimizing pore structures to balance efficient electrolyte utilization with high capacity, especially under practical conditions requiring lean electrolyte and high‐power performance.

We next examined the impact of optimized porosity on the cycling performance of electrodes under lean electrolyte and high areal capacity conditions. For the cycling experiments, we set a capacity of 4 mAh cm^−2^ and used two levels of very lean electrolyte loading, corresponding to EL/C of 6 and 4. The electrolyte used for the cycling experiments was 0.5 m LiTFSI + 0.5 m lithium nitrate (LiNO_3_) + 0.2 m lithium bromide (LiBr) in TEGDME. Figure 4d,e depicts the galvanostatic discharge/charge profiles of the GMS and iGMS electrodes, respectively, for selected cycles with an EL/C ratio of 6. The XRD patterns of the discharged and charged electrodes in Figures S21–S24 (Supporting Information) confirm the deposition and decomposition of Li_2_O_2_ as the discharge product. As shown in Figure 4f, the GMS and iGMS electrodes demonstrated 24 and 23 stable cycles, respectively at an EL/C ratio of 6. Similarly, Figure S25 (Supporting Information) shows that the KB electrode achieved 19 cycles under similar conditions. XRD patterns in Figures S26 and S27 (Supporting Information) show the evidence of Li_2_O_2_ deposition and decomposition during discharge and recharge of the KB electrode. With the progress of cycling, all cells exhibited a gradual increase in voltage polarization and capacity fade, which are typical degradation phenomena in LOBs caused by the accumulation of insulating side products that increase impedance and clog the electrode pores. The slightly higher cycling stability of GMS‐based electrodes compared to KB can be attributed to the better crystallinity and higher degrees of graphitization of GMS. A significant difference in stability emerged when the electrolyte loading was reduced to an EL/C of 4. Under this condition, the LOB cell with GMS electrode could not even charge during the first cycle, and KB electrode failed within three cycles, as shown in Figure 4g, and Figure S28 (Supporting Information), respectively. These failures were marked by a sudden voltage spike during charging, indicating a sharp rise in cell impedance likely due to electrolyte diffusion limitation. In contrast, the LOB cell with iGMS electrode, in Figure 4h, exhibited stable cycling for 23 cycles, even with the low EL/C of 4 (which is equal to the capacity normalized value of 3.25 g Ah^−1^). The cycling performance comparison of GMS and iGMS electrodes at an EL/C ratio of 4 (Figure 4i) clearly highlights that pore interconnectivity plays a critical role, beyond the contribution of pore volume alone, in determining long‐term cell stability, particularly with lean amount of electrolyte. The interconnected porous network and reduced pore volume of the iGMS electrode likely enhanced the diffusion and retention of the electrolyte within the electrode pores, particularly under extremely lean electrolyte conditions. As a result, the iGMS electrode demonstrated the most stable cycling with the lowest electrolyte loading.

### 2.4 High‐Rate Cycling of LOBs

As mentioned earlier, the rate performance of LOBs is governed by the efficiency of O_2_ diffusion within the positive electrode and the ionic conductivity of the electrolyte. Given the highly interconnected pore structure of the iGMS electrode, designed for higher electrolyte and O_2_ diffusion and minimal electrolyte requirement, a high‐rate capability is expected. To confirm this, we compared the discharge/charge performances of cells using GMS, iGMS, and KB electrodes at a high current density of 1.5 mA cm^−2^. The tests employed a lean electrolyte with a low EL/C ratio of 5. As shown in Figure 4j, the LOB cell with GMS electrode failed to discharge even in the first cycle under these demanding conditions. Similarly, the cell with KB electrode (Figure S29, Supporting Information) failed to complete the charge process during the first cycle, prematurely reaching the cut‐off voltage of 4.5 V vs Li/Li⁺. These sudden voltage polarizations in the cases of GMS and KB electrodes indicate high cell impedance, which makes high‐rate cycling impossible. In contrast, the cell with iGMS electrode successfully cycled for 90 cycles at the same high current density and low electrolyte loading. The discharge/charge voltage profiles for selected cycles, presented in Figure 4k, illustrate the stable performance of iGMS electrode. This finding of better rate‐capability of iGMS electrode confirms the benefits of interconnected pores and proper pore filling under lean electrolyte conditions. Figure 4l compares the high‐power cycling stability of the iGMS electrode with previously reported electrodes cycled with a minimum current density of 0.8 mA cm^−2^.^[^ ^38^, ^39^ ^]^ First of all, the number of reports on high‐rate cycling of LOB is very limited. Moreover, none of the prior designs could achieve any significant cycling stability at high rate. In contrast, the iGMS electrode achieved an impressive 90 cycles at a high current density of 1.5 mA cm^−2^ under lean electrolyte conditions, setting a new benchmark for high‐power LOBs.

### 2.5 Analysis of Cell Failure

**Figure 5:**
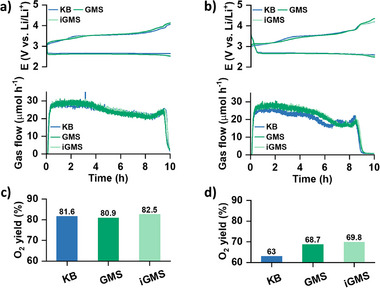
Galvanostatic discharge/charge profiles and O_2_ evolution rates during charging for the a) 1^st^ and b) 5^th^ cycles using KB, GMS, and iGMS electrodes. Comparison of O_2_ yield in different electrodes for the c) 1^st^ and d) 5^th^ cycles.

Alongside electrolyte depletion, the degradation of both the electrode and the electrolyte plays a critical role in determining the cycle life of LOBs.^[^ ^40^, ^41^ ^]^ To investigate this, we conducted online electrochemical mass spectrometry (online MS) to quantify gas evolution during cell charging, providing insights into the relative stabilities of the electrodes. The fundamental operation of LOBs relies on the reversible electrochemical reduction of O_2_ during discharge and its evolution during charging. As a result, monitoring O_2_ evolution is a key metric for evaluating the reversibility of LOB processes. However, alongside the expected O_2_ evolution, undesirable parasitic reactions involving both the electrode and the electrolyte can lead to the release of CO_2_. Consequently, analyzing the gases generated during cycling provides valuable insights into the reactions occurring at the positive electrode and their influence on the stability of the cells. We employed a specialized two‐compartment cell design for the online MS experiments. This setup isolates the Li negative electrode from the positive electrode using a glass ceramic separator, allowing for the exclusive detection of gases evolved from the positive electrode. We compared the gas evolution trends of KB, GMS, and iGMS electrodes to evaluate the stability of these carbon materials. **Figure** **5**a,b presents the voltage profiles for the 1st and 5th cycles of LOBs using these electrodes with a limited capacity of 4 mAh cm^−2^, along with the corresponding gas evolution rates. The quantitative results for O_2_ evolution are summarized in Figure 5c,d. During the 1st charge cycle, there was no significant difference in the evolution rates of O_2_ between the LOB cells with KB and two GMS‐based electrodes (Figure 5c). However, by the 5th charge cycle (Figure 5d), a noticeable decrease in O_2_ evolution was observed in the cell with KB electrode compared to the cell with GMS electrodes. Specifically, the KB electrode exhibited 63% O_2_ evolution, while both the GMS electrodes achieved about 69% O_2_ yield. The O_2_ yield is calculated based on the oxygen consumption rate of 2e^−^ per O_2_ molecule during discharge. Since the same electrolyte was used and a consistent cut‐off voltage was employed, the difference in the O_2_ evolution can be attributed to the differences in the stabilities of the electrodes. As shown by the physicochemical characterizations in Figures S3–S10 (Supporting Information), the GMS carbon exhibits a higher degree of graphitization, fewer defects, and a greater carbon content than the KB carbon. The more ordered carbon framework, with reduced surface functional groups and defect sites, is less prone to parasitic reactions with reactive oxygen species such as singlet oxygen and superoxide intermediates. This structural stability improves the chemical robustness of the carbon matrix during repeated discharge/charge cycles, mitigating the formation of irreversible carbonates and other degradation products. These intrinsic advantages of the GMS carbon account for its enhanced O_2_ yield. Notably, optimization of the electrode pore architecture does not appear to significantly influence the O_2_ reversibility.

## 3 Conclusion

This study highlights the pivotal role of an interconnected porous network and optimized porosity in carbon electrodes for achieving high capacity, long cycling life, and stable performance under high‐power operations in LOBs operating with lean electrolyte conditions. While increasing electrode porosity is commonly associated with higher capacity, proper connection of the porous network is crucial to ensure efficient diffusion of electrolyte and O_2_, which are essential for high‐power performance. Additionally, electrodes with unoptimized large pore volumes often require higher electrolyte volumes to maintain stable cycling, offsetting the specific capacity benefits. Conversely, upon lowering the electrolyte volume, highly porous electrodes face severe electrolyte depletion, leading to pronounced voltage polarization and premature cell failure. The development of electrodes with a highly interconnected pore network and optimized pore volume, minimizing inactive porosity, offers a promising pathway to overcoming the energy‐power trade‐off in LOBs. For example, a GMS electrode with an optimized pore structure demonstrated a high discharge capacity of 2520 mAh g^−1^ at a discharge rate of 1.0 mA cm^−2^ under a low electrolyte loading ratio (EL/C) of 5. Furthermore, this pore‐optimized GMS electrode achieved stable cycling at a high areal capacity of 4 mAh cm^−2^ under an EL/C ratio of 4 and delivered exceptional high‐power cycling performance, sustaining 90 cycles at a high rate of 1.5 mA cm^−2^. In contrast, electrodes with greater pore volumes but lacking proper pore connectivity exhibited inferior capacity, reduced cycling stability, and lower rate‐capability under comparable lean electrolyte conditions. These findings underscore the necessity of finely tuned electrode pore structures to balance capacity, cycling stability, rate performance, and electrolyte utilization in LOBs. They also offer valuable insights into the design of advanced carbon materials, paving the way for the development of next‐generation practical lithium‐oxygen batteries.

## Conflict of Interest

The authors declare no conflict of interest.

## Supporting information



Supporting Information

## Data Availability

The data that support the findings of this study are available from the corresponding author upon reasonable request.
