# Supplementary material for: Interconnected Hierarchically Porous Graphene‐Based Membrane Electrode for High‐Power and Long‐Cycle Lithium–Oxygen Battery
Source: Adv Sci (Weinh). 2025 Dec 1;13(9):e19091. doi: 10.1002/advs.202519091 (PMC12904046; doi:10.1002/advs.202519091)
Supplement: Supplementary file 1 — Supporting Information [file ADVS-13-e19091-s001.pdf]

Supporting Information

Interconnected Hierarchically Porous Graphene-based Membrane Electrode for High-Power and Long-Cycle Lithium-Oxygen Battery

Arghya Dutta, Takashi Kameda, Taiga Ozawa, Anna Myojin, Minako Nishioka, Wei Yu, Hirotomo Nishihara, and Shoichi Matsuda**

Methods

1. Simulation of oxygen (O_2) diffusion in the electrode under different conditions

The variation of the effective diffusion coefficient (D_{eff}) of O_2 with porosity (ε) and tortuosity (τ) was calculated using the following equation

$$D_{eff} = D_0 \frac{\varepsilon}{\tau}$$

The value of bulk diffusion coefficient (D_0) of O_2 in tetraethylene glycol dimethyl ether (TEGDME) based electrolyte with a Li-salt concentration of 1 M was approximated to be 2.0 × 10^{-5} cm^2 s^{-1} based on previous reports.[1,2]

The contour plots of the normalized O_2 concentration across the electrode thickness at different current densities were calculated using the equation

$$C(x) = C_0 \, exp\left(-x \frac{j}{nFC_0D_{eff}}\right)$$

The bulk concentration (C_0) of O_2 in TEGDME-based electrolyte with a Li-salt concentration of 1 M was approximated to be 4.0 mM based on a previous report.[3]

2. Synthesis of graphene mesosponge (GMS) carbon

The GMS carbon was synthesized using Al_2O_3 nanoparticles (SBa-200, Sasol) as the template. A carbon layer was deposited onto the Al_2O_3 nanoparticles by chemical vapor deposition (CVD) under a CH_4/Ar atmosphere (20 vol.% CH_4, 900 °C). Subsequently, the Al_2O_3 core was selectively etched away by immersing the composite in hydrofluoric acid (HF), leaving behind a porous carbon framework. Finally, the obtained carbon material was subjected to heat treatment at 1800 °C for 1 h under reduced pressure (Ar, 10 Pa) to yield the GMS sample.

3. Fabrication of the self-standing GMS membrane electrode

The self-standing carbon gel-based membranes were developed by using GMS powder samples. The electrode fabrication process involved the following steps:

3.1. Slurry preparation

The process begins with preparing a slurry composed of GMS powder as the primary electrode material, single-walled carbon nanotubes (OCSiAl, TUBALL, average diameter ~1.6 nm, average length ~5 μm) as a secondary carbon source to enhance the mechanical stability of the membrane, polyacrylonitrile (PAN) and/or polyethylene oxide (PEO) as polymeric components to optimize macroporosity via the non-solvent-induced phase separation (NIPS) method, and N-methyl-2-pyrrolidone (NMP) as the solvent to ensure uniform dispersion.

3.2. Film formation

The slurry was cast onto a substrate using the doctor blade method to produce a uniform film with controlled thickness.

3.3. Pore generation

The cast film was subsequently immersed in methanol, a nonsolvent, to induce phase separation and form a porous structure via the NIPS process.

3.4. Drying and stabilization of the film

The obtained film was dried at 80 °C for 10 h to remove residual solvent, followed by infusibilization at 230 °C for 3 h in an air-circulating oven (DN411, Yamato Scientific Co., Ltd.).

3.5. Carbonization

Carbonization was performed in a box furnace (Denken High Dental Co., Ltd.) under a nitrogen atmosphere (800 mL min^{-1}). The temperature was ramped to 1050 °C at 10 °C min^{-1}, held for 3 h, and then cooled naturally to room temperature.

The same procedure was followed to prepare the Ketjenblack (KB) membranes. In the case of KB membrane, only PAN was used during NIPS process.

4. Characterization

The pore structures of the membranes were evaluated by nitrogen adsorption–desorption measurements (3 FLEX, Micromeritics), and macropore size distributions were determined by mercury intrusion porosimetry (Autopore IV 9505, Shimadzu). The micropore (< 2 nm), small mesopore (< 20 nm), and large mesopore (> 20 nm) volumes were analyzed by Horvath-Kawazoe (H-K), Density Functional Theory (DFT), and Barrett-Joyner-Halenda (BJH) methods, respectively. X-ray diffraction patterns were analyzed by an X-ray diffractometer (SmartLab, Rigaku). A hermetically sealed sample holder was used for the XRD measurements of the cycled electrodes. Morphological features were examined with field-emission scanning electron microscopy (FE-SEM, S-4800, Hitachi). The degree of graphitization was assessed using Raman spectroscopy (RamanTouch-VIS-NIR, Nanophoton). Surface chemical composition was analyzed by X-ray photoelectron spectroscopy (XPS, VersaProbe II, ULVAC-PHI). Transmission electron microscopy (TEM, JEM-ARM200F, JEOL) was used for detailed structural imaging of GMS sample. Thermogravimetric analyses (TGA, TG-DTA8122HMS, Rigaku) of PAN and PEO were carried out under helium (He) atmosphere with a heating rate of 5 °C min^{-1}.

5. Lithium–Oxygen cell assembly and discharge performance test

The electrolyte was prepared by dissolving 1.0 M lithium bis(trifluoromethanesulfonyl)imide (LiTFSI, >99.9%, Kishida Chemical Co., Ltd.) in tetraethylene glycol dimethyl ether (TEGDME, >99%, Kishida Chemical Co., Ltd.). The water content was confirmed to be below 30 ppm by Karl Fischer titration. Self-standing carbon membranes, vacuum-dried at 100 °C for 12 h, were employed as cathodes. Lithium–oxygen cells with an active area of 2 × 2 cm^2 were assembled in a dry room (<10 ppm H_2O). The cell configuration consisted of lithium foil (Honjo Metal Co., Ltd.) as the negative electrode, a polyolefin separator, the carbon positive electrode, and a gas diffusion layer (TGP-H-060, Toray, Japan) stacked in sequence. Electrolyte impregnation was carried out using the vacuum impregnation method to fill ~80% of the electrode pore volume. A spring coil was used to apply 100 kPa pressure during cell assembly. The cells were operated in an oxygen (O_2) atmosphere with a continuous O_2 flow of 80 mL min^{-1}. Electrochemical measurements were performed using a battery tester (SD8, Hokuto Denko Corp.), with discharge carried out at 0.4 and 1.0 mA cm^{-2} to a cutoff voltage of 2.0 V vs. Li/Li^+.

6. Lithium–Oxygen cell assembly and discharge/charge cycling test

For cycling experiments, the electrolyte consisted of 0.5 M lithium bis(trifluoromethanesulfonyl)imide (LiTFSI, >99.9%, Kishida Chemical Co., Ltd.), 0.5 M lithium nitrate ($LiNO_3$, 99.99% trace metals, Sigma–Aldrich), and 0.2 M lithium bromide (LiBr, 99.995% trace metals, Sigma–Aldrich) dissolved in tetraethylene glycol dimethyl ether (TEGDME, > 99%, Kishida Chemical Co., Ltd.). $LiNO_3$ and LiBr were pre-dried under vacuum at 120 °C for over 3 days. Water content of the electrolyte was below 30 ppm as measured by Karl Fischer titration.

Cells followed the same configuration as the discharge tests, except the lithium-metal anode was protected by a ceramic-based solid-state separator (LICGC, 0.50 mm, Ohara, Inc.) placed between polyolefin separators. Electrolyte was introduced into the carbon cathodes via vacuum impregnation, with two electrolyte-to-carbon mass ratios (EL/C) of 6 and 4. Electrochemical cycling was performed using a battery tester (TOSCAT, Toyo System Co., Ltd.), with capacity limited to 4.0 mAh cm^{-2} and voltage cut-off of 2.0–4.5 V vs. Li/Li^+ at 0.4 mA cm^{-2}. High-rate tests used 0.5 mAh cm^{-2} at 1.5 mA cm^{-2}. All cells were operated in an O_2-filled chamber with continuous O_2 flow of 80 mL min^{-1}.

7. Online Mass spectroscopic (MS) analysis

High-resolution electrochemical mass spectrometry (MS) was performed using an MS system (M-401GA, CANON ANELVA Corp.) in an online configuration. An electrochemical flow

cell with an internal volume of ~24 mL (70 mm diameter, 15 mm depth) was employed, containing the same electrode components as the previously described lithium–oxygen cells. The negative and positive electrodes were separated by a glass-ceramic separator. The positive electrode side used the ternary-salt electrolyte, while the negative electrode was filled with 4 M lithium bis(fluorosulfonyl)imide (LiFSI) in dimethoxyethane (DME). Before measurement, the cell was purged with O_2 and discharged to a capacity of 4 mAh cm^{-2}. Excess O_2 was then removed by flushing with helium (50 mL min^{-1}) for 1 min. During charging, evolved gases were continuously carried by helium at 5 mL min^{-1} to the MS detector. Gas transfer to the MS was achieved through a capillary tube (0.05 mm internal diameter, 7 m length).

8. Impedance analysis of symmetric cells

The impedance of Carbon|Separator|Carbon symmetric cells incorporating GMS and iGMS membrane electrodes was measured using a Biologic VMP3. The electrolyte consisted of 0.5 M LiTFSI, 0.5 M $LiNO_3$, and 0.2 M LiBr dissolved in tetraglyme. The measurements were performed over a frequency range of 1 MHz to 1 mHz with an applied voltage amplitude of 5 mV.

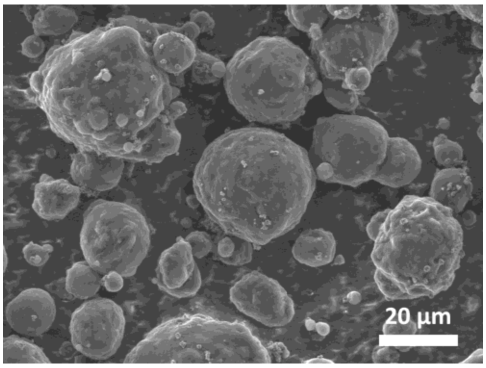

Figure S1. SEM image of GMS powder sample.

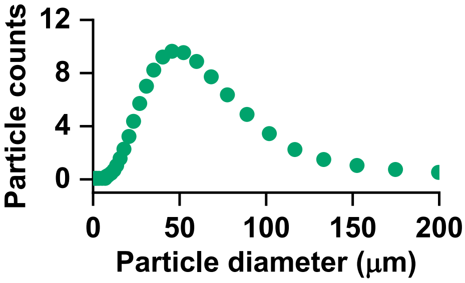

Figure S2. Particle-size distribution curve of GMS powder estimated from SEM analysis.

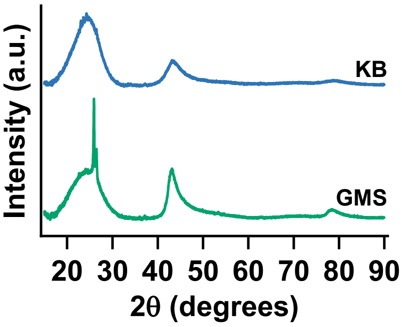

Figure S3. XRD patterns of KB and GMS powder samples. The sharp (002) peak in the GMS sample comes from graphite contamination during the synthesis.

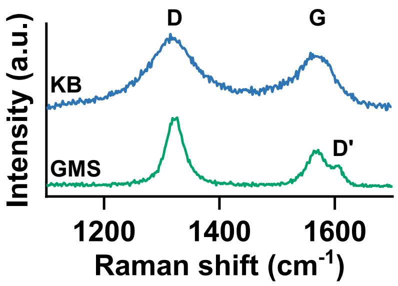

Figure S4. Raman spectra of KB and GMS powder samples.

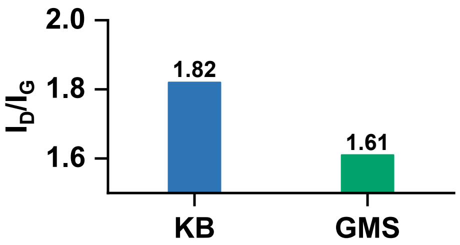

Figure S5. I_D/I_G ratio of KB and GMS powder samples estimated from the Raman spectra of the respective samples.

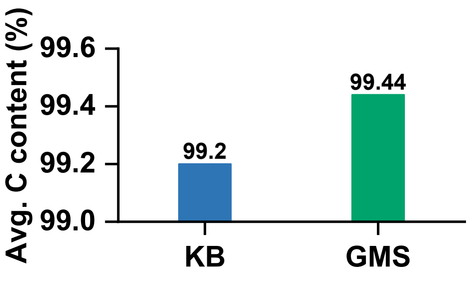

Figure S6. Estimation of carbon contents in KB and GMS powders from XPS measurements.

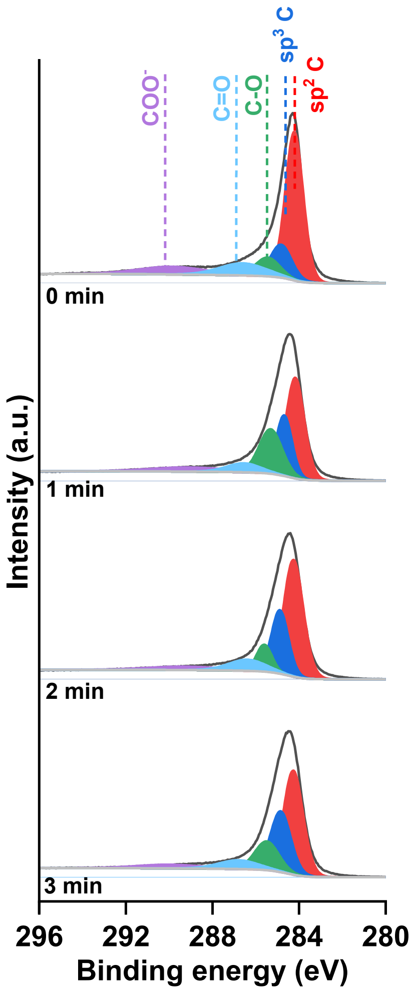

Figure S7. C1s XPS spectra of KB powder sample measured after Ar etching for different durations.

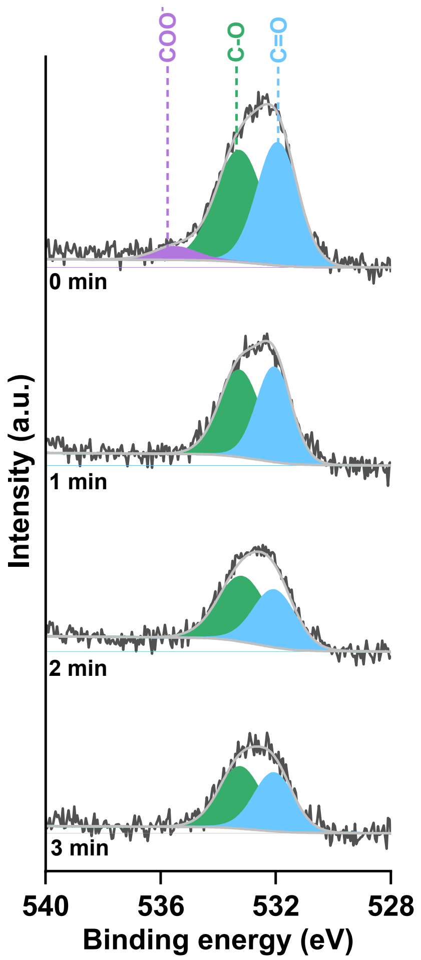

Figure S8. O1s XPS spectra of KB powder sample measured after Ar etching for different durations.

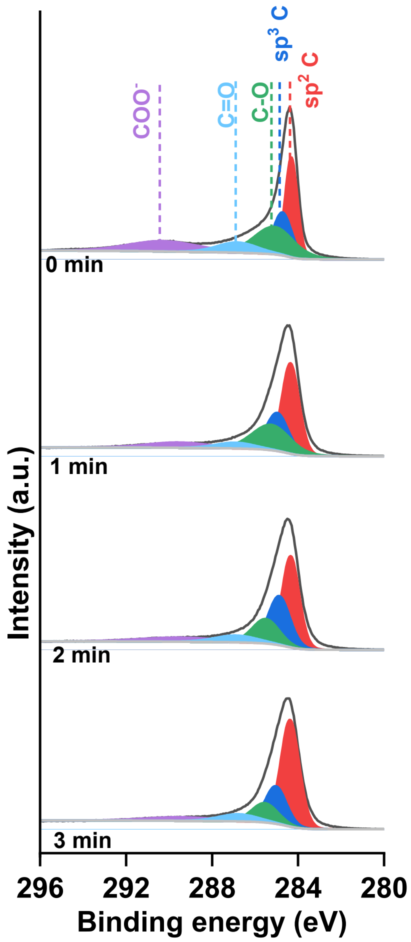

Figure S9. C1s XPS spectra of GMS powder sample measured after Ar etching for different durations.

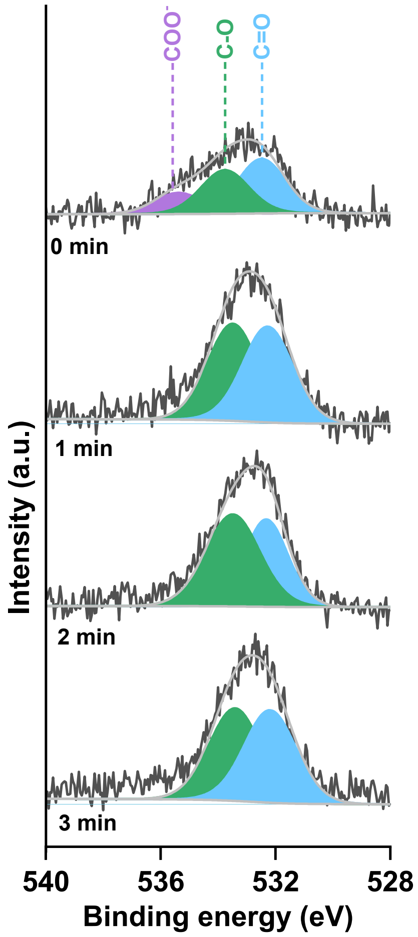

Figure S10. O1s XPS spectra of GMS powder sample measured after Ar etching for different durations.

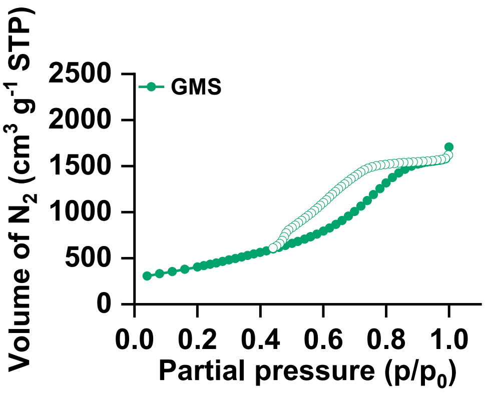

Figure S11. N_2 adsorption/desorption isotherm of GMS membrane measured at -196 °C. The volume of N_2 is normalized to the total mass of the membrane.

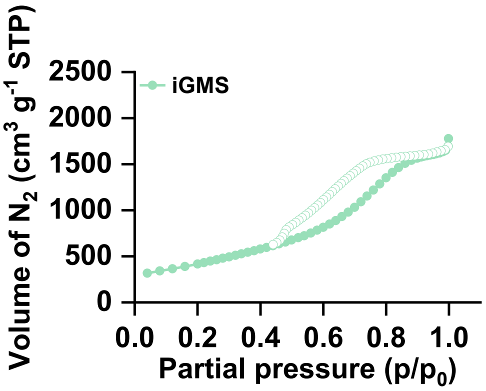

Figure S12. N_2 adsorption/desorption isotherm of iGMS membrane measured at -196 °C. The volume of N_2 is normalized to the total mass of the membrane.

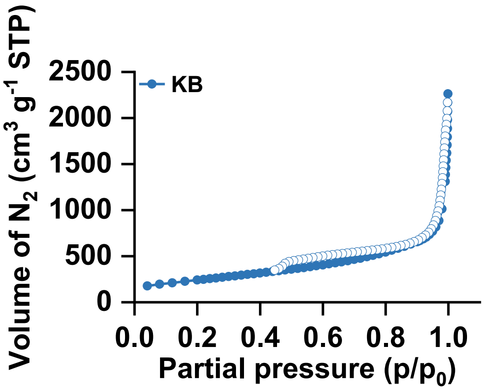

Figure S13. N_2 adsorption/desorption isotherm of iGMS membrane measured at -196 °C. The volume of N_2 is normalized to the total mass of the membrane.

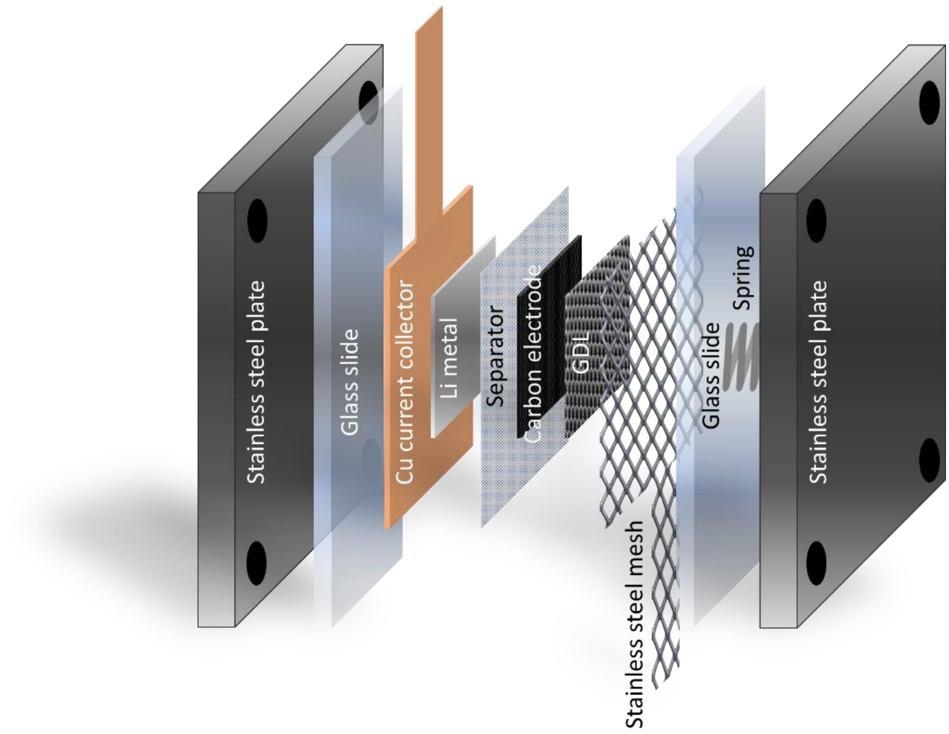

Figure S14. Schematic representation of the stack-type cell used in this study.

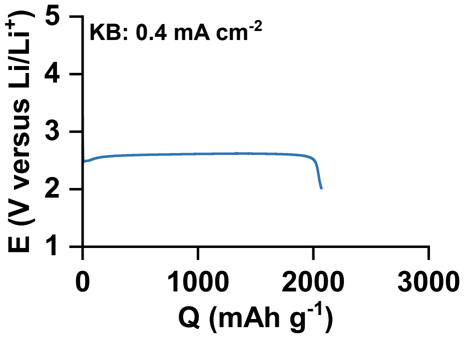

Figure S15. Galvanostatic discharge profile of LOB cell with KB electrode at 0.4 mA cm^{-2}.

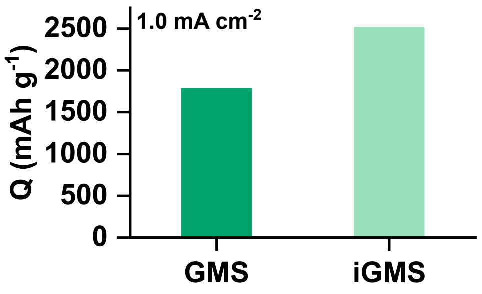

Figure S16. Comparison of discharge capacities of GMS and iGMS electrodes at 1.0 mA cm^{-2}.

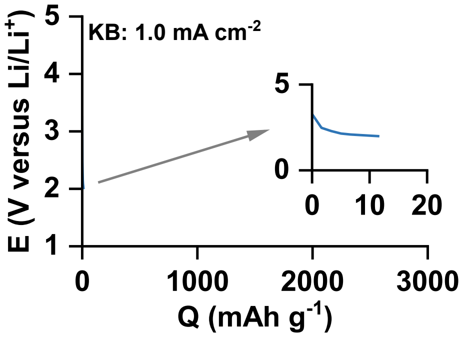

Figure S17. Galvanostatic discharge profile of LOB cell with KB electrode at 1.0 mA cm^{-2}.

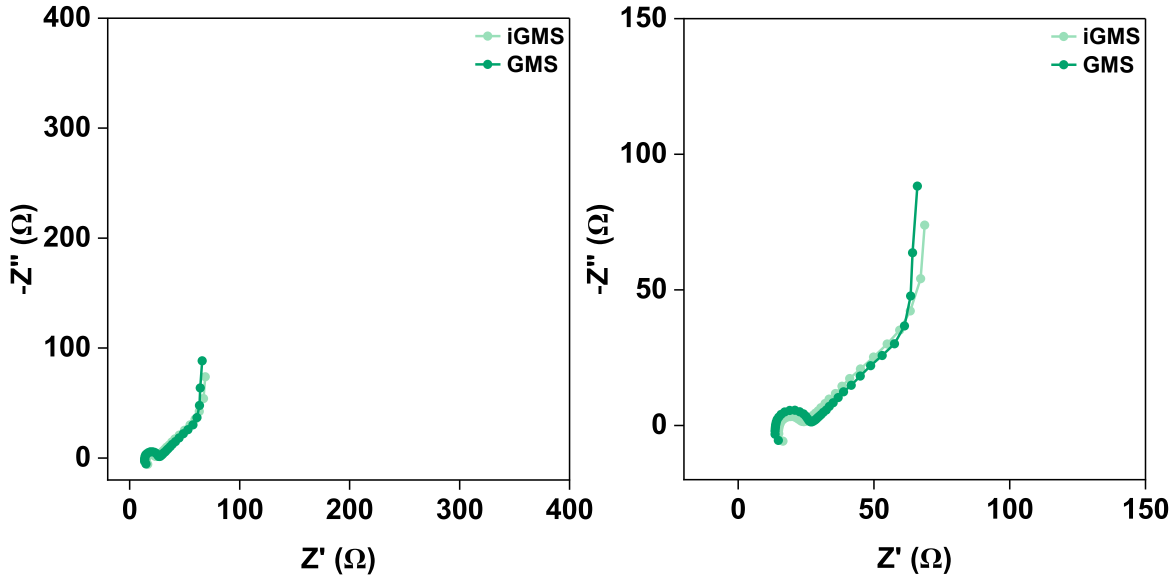

Figure S18. Nyquist plots of Carbon|Separator|Carbon symmetric cell with an electrolyte loading amount of EL/C = 6. The right panel shows a magnified view of the left graph.

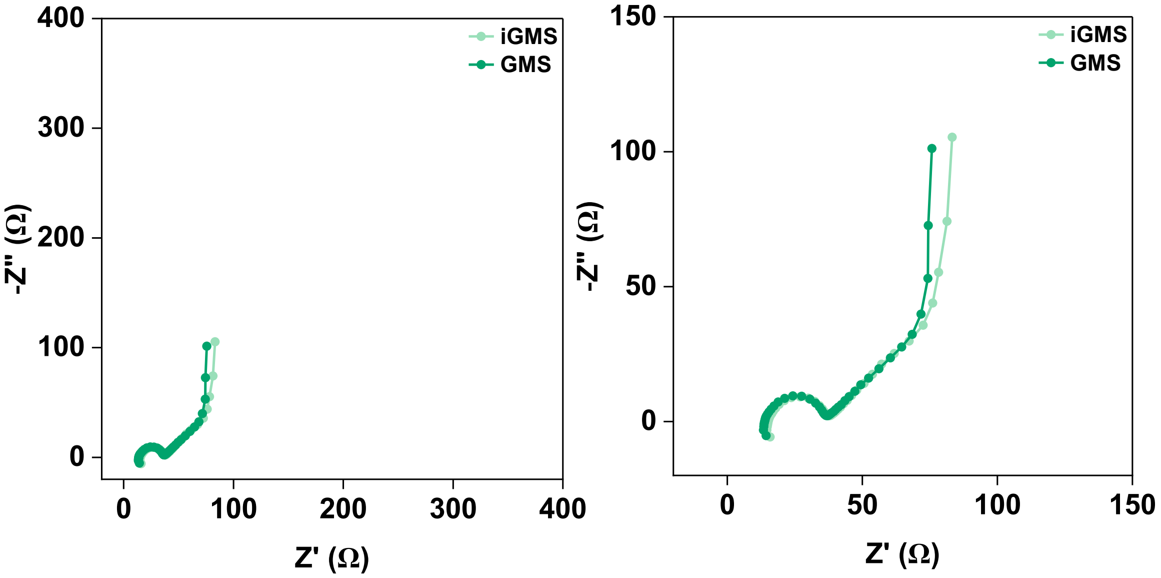

Figure S19. Nyquist plots of Carbon|Separator|Carbon symmetric cell with an electrolyte loading amount of EL/C = 5. The right panel shows a magnified view of the left graph.

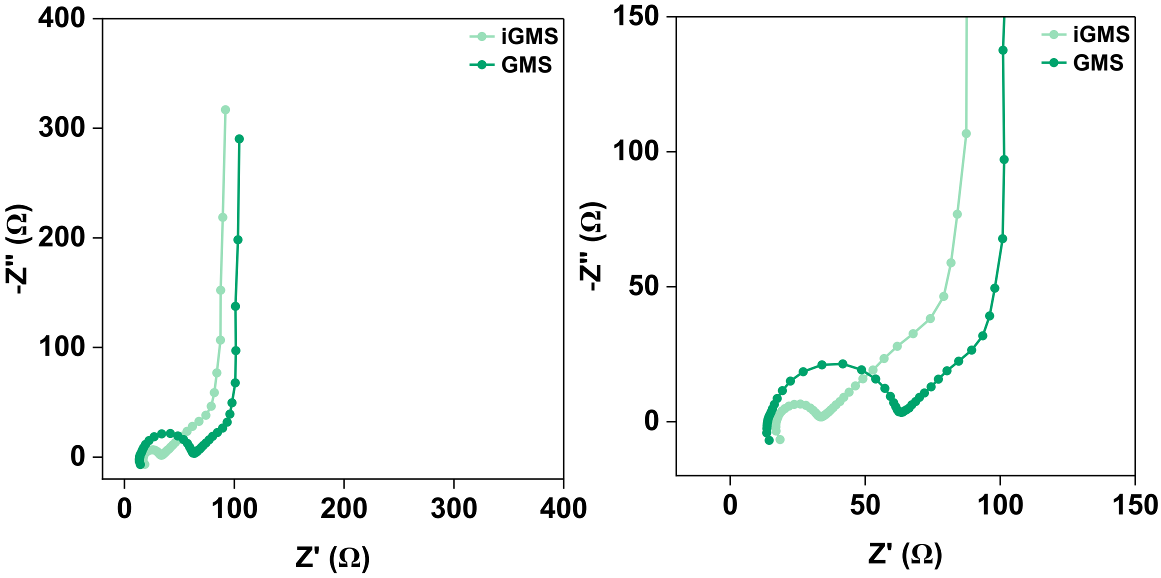

Figure S20. Nyquist plots of Carbon|Separator|Carbon symmetric cell with an electrolyte loading amount of EL/C = 4. The right panel shows a magnified view of the left graph.

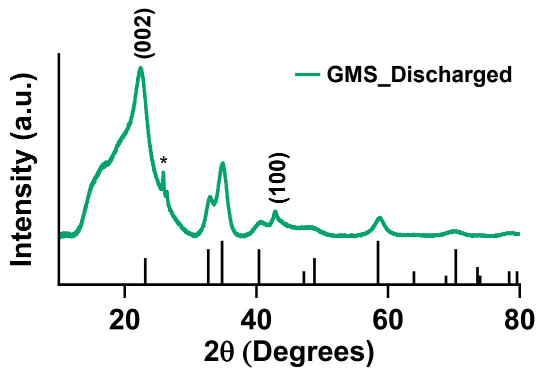

Figure S21. XRD pattern of the discharged GMS electrode. The reference diffraction peaks of standard Li_2O_2 are shown as bars at the bottom. The characteristic (002) and (100) peaks of GMS carbon, along with the (002) graphite impurity peak (marked with an asterisk), are also indicated.

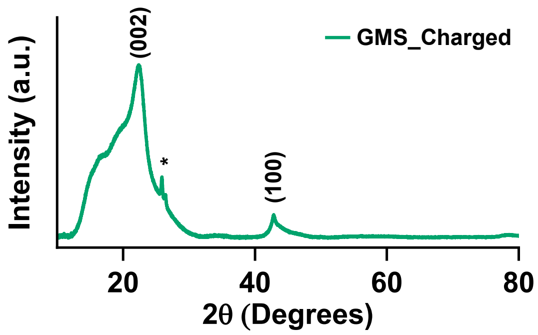

Figure S22. XRD pattern of the charged GMS electrode. The characteristic (002) and (100) peaks of GMS carbon, along with the (002) graphite impurity peak (marked with an asterisk), are shown.

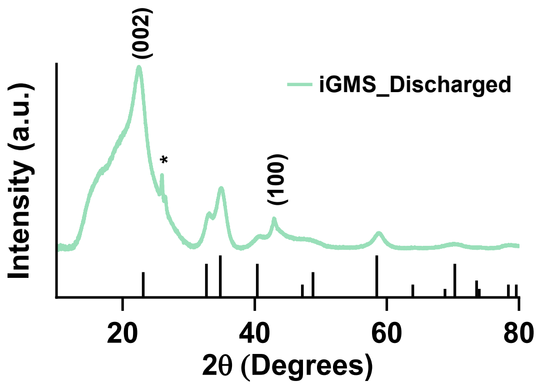

Figure S23. XRD pattern of the discharged iGMS electrode. The reference diffraction peaks of standard Li_2O_2 are shown as bars at the bottom. The characteristic (002) and (100) peaks of iGMS carbon, along with the (002) graphite impurity peak (marked with an asterisk), are also indicated.

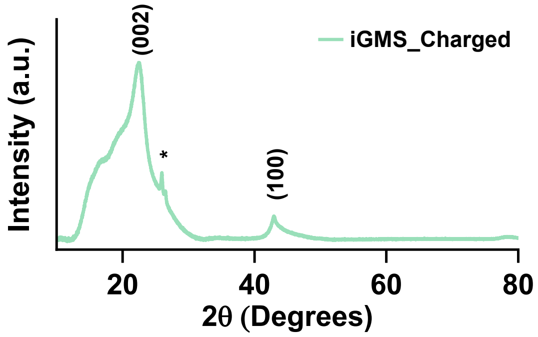

Figure S24. XRD pattern of the charged iGMS electrode. The characteristic (002) and (100) peaks of iGMS carbon, along with the (002) graphite impurity peak (marked with an asterisk), are shown.

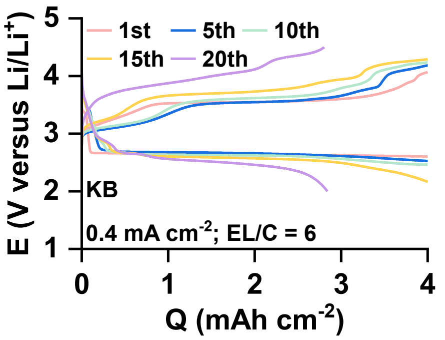

Figure S25. Cycling profile of KB at 0.4 mA cm^{-2} with 4 mAh cm^{-2} capacity under EL/C = 6.

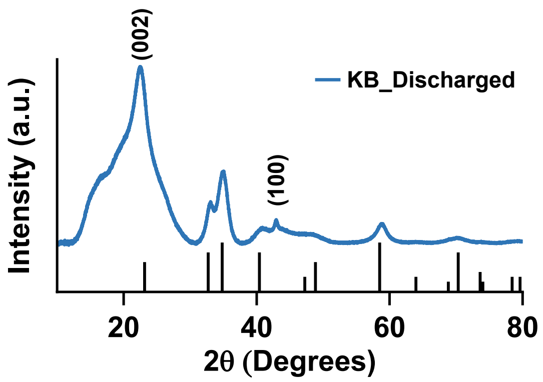

Figure S26. XRD pattern of the discharged KB electrode. The reference diffraction peaks of standard Li_2O_2 are shown as bars at the bottom. The characteristic (002) and (100) peaks of KB carbon are also indicated.

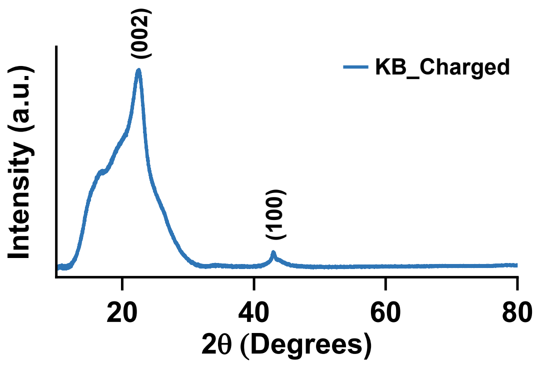

Figure S27. XRD pattern of the charged KB electrode. The characteristic (002) and (100) peaks of KB carbon are shown.

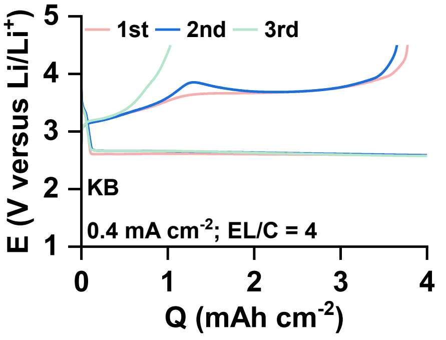

Figure S28. Cycling profile of KB at 0.4 mA cm^{-2} with 4 mAh cm^{-2} capacity under EL/C = 4.

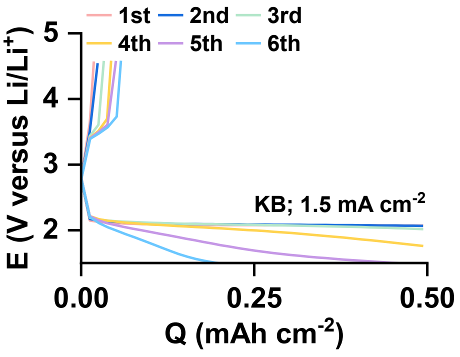

Figure S29. High-rate cycling of KB electrode.

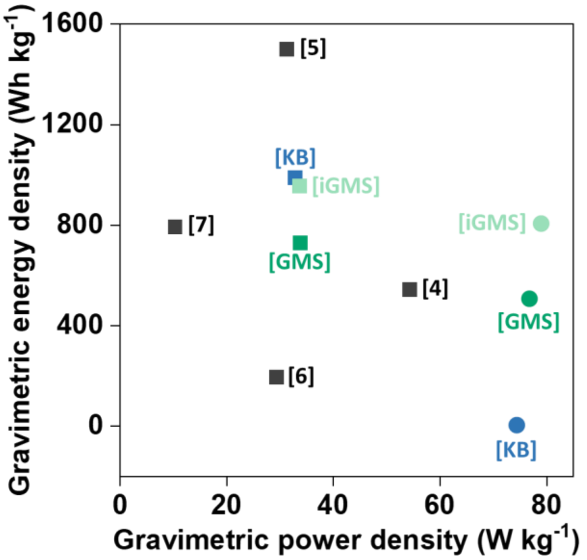

Figure S30. Gravimetric energy density values of various electrodes under different power density values.

Table S1. Mass loading, mass normalized current densities, and specific capacities of different membrane electrodes.

Sample	Carbon loading (mg cm^{-2})	Areal current density (mA cm^{-2})	Mass normalized current density (mA g^{-1})	Absolute capacity (mAh)	Specific capacity (mAh g^{-1})
KB	5.08	0.4	78.82	48.2	2374.38
iGMS	3.88	0.4	103.09	45.37	2923.32
GMS	3.58	0.4	111.89	34.5	2412.59
KB	4.68	1	213.90	0.23	12.30
iGMS	3.88	1	257.73	40.8	2628.87
GMS	3.58	1	279.72	26.38	1844.76

Table S2. Comparison of energy and power densities of different electrodes measured in this study along with the electrodes reported in the literature.

Sample /Ref.	Areal current density (mA cm^{-2})	Areal capacity (mAh cm^{-2})	Areal mass of the cell (mg cm^{-2})	Average discharge voltage (V)	Gravimetric energy density (Wh kg^{-1})	Gravimetric power density (W kg^{-1})
KB	0.4	12.05	30.10	2.47	988.98	32.82
iGMS	0.4	11.3425	31.12	2.62	954.92	33.67
GMS	0.4	8.625	30.97	2.62	729.77	33.84
KB	1	0.0575	28.20	2.1	4.28	74.48
iGMS	1	10.2	31.12	2.46	806.29	79.04
GMS	1	6.595	30.97	2.38	506.89	76.86
[4]	0.3	3	14.85	2.69	543.4	54.34
[5]	0.4	19.21	33.04	2.58	1500.64	31.23
[6]	1.5	10	138.09	2.7	195.5	29.33
[7]	0.4	30.8	103	2.65	793.0	10.3

The mass of the cell includes masses of the electrodes, gas diffusion layer, current collectors, separator, and electrolyte.

References

[1] A. Schürmann, R. Haas, M. Murat, N. Kuritz, M. Balaish, Y. Ein-Eli, J. Janek, A. Natan, D. Schröder, Diffusivity and Solubility of Oxygen in Solvents for Metal/Oxygen Batteries: A Combined Theoretical and Experimental Study. *J. Electrochem. Soc.* **2018**, *165*, A3095.

[2] R. Haas, M. Murat, M. Weiss, J. Janek, A. Natan, D. Schröder, Understanding the Transport of Atmospheric Gases in Liquid Electrolytes for Lithium–Air Batteries. *J. Electrochem. Soc.* **2021**, *168*, 070504.

[3] C. O. Laoire, S. Mukerjee, K. M. Abraham, E. J. Plichta, M. A. Hendrickson, Influence of Nonaqueous Solvents on the Electrochemistry of Oxygen in the Rechargeable Lithium−Air Battery. *J. Phys. Chem. C* **2010**, *114*, 9178.

[4] S. Matsuda, E. Yasukawa, T. Kameda, S. Kimura, S. Yamaguchi, Y. Kubo, K. Uosaki, Carbon-black-based self-standing porous electrode for 500 Wh/kg rechargeable lithium-oxygen batteries. *Cell. Rep. Phys. Sci.* **2021**, *2*, 100506.

[5] A. Dutta, T. Kameda, J. Takada, Y. Nakajima, T. Morishita, S. Matsuda, Quantitative Porosity Engineering of Carbon Electrode in Lithium–Oxygen Batteries with Cell-Level Gravimetric Energy Density Over 1500 Wh kg^{-1}. *Adv. Sci.* **2025**, e14406. DOI: 10.1002/advs.202514406

[6] Y. J. Lee, S. H. Park, S. H. Kim, Y. Ko, K. Kang, Y. J. Lee, High-Rate and High-Areal-Capacity Air Cathodes with Enhanced Cycle Life Based on RuO_2/MnO_2 Bifunctional Electrocatalysts Supported on CNT for Pragmatic Li–O_2 Batteries. *ACS Catal.* **2018**, *8*, 2923

[7] W. Yu, Z. Shen, T. Yoshii, S. Iwamura, M. Ono, S. Matsuda, M. Aoki, T. Kondo, S. R. Mukai, S. Nakanishi, H. Nishihara, Hierarchically Porous and Minimally Stacked Graphene Cathodes for High-Performance Lithium–Oxygen Batteries. *Adv. Energy Mater.* **2024**, *14*, 2303055.
